# Untargeted NMR Metabolomics Reveals Alternative Biomarkers and Pathways in Alkaptonuria

**DOI:** 10.3390/ijms232415805

**Published:** 2022-12-13

**Authors:** Daniela Grasso, Michela Geminiani, Silvia Galderisi, Gabriella Iacomelli, Luana Peruzzi, Barbara Marzocchi, Annalisa Santucci, Andrea Bernini

**Affiliations:** 1 Department of Biotechnology, Chemistry and Pharmacy, University of Siena, Via A, Moro 2, 53100 Siena, Italy; 2 Centro Regionale Medicina di Precisione, 53100 Siena, Italy; 3 ARTES 4.0, 56025 Pontedera, Italy

**Keywords:** rare diseases, alkaptonuria, ochronosis, phenylalanine and tyrosine metabolism, serine, glycine, and one-carbon metabolism, TCA cycle, trace amines, untargeted metabolomics, nuclear magnetic resonance

## Abstract

Alkaptonuria (AKU) is an ultra-rare metabolic disease caused by the accumulation of homogentisic acid (HGA), an intermediate product of phenylalanine and tyrosine degradation. AKU patients carry variants within the gene coding for homogentisate-1,2-dioxygenase (HGD), which are responsible for reducing the enzyme catalytic activity and the consequent accumulation of HGA and formation of a dark pigment called the ochronotic pigment. In individuals with alkaptonuria, ochronotic pigmentation of connective tissues occurs, leading to inflammation, degeneration, and eventually osteoarthritis. The molecular mechanisms underlying the multisystemic development of the disease severity are still not fully understood and are mostly limited to the metabolic pathway segment involving HGA. In this view, untargeted metabolomics of biofluids in metabolic diseases allows the direct investigation of molecular species involved in pathways alterations and their interplay. Here, we present the untargeted metabolomics study of AKU through the nuclear magnetic resonance of urine from a cohort of Italian patients; the study aims to unravel molecular species and mechanisms underlying the AKU metabolic disorder. Dysregulation of metabolic pathways other than the HGD route and new potential biomarkers beyond homogentisate are suggested, contributing to a more comprehensive molecular signature definition for AKU and the development of future adjuvant treatment.

## 1. Introduction

**Figure 1:**
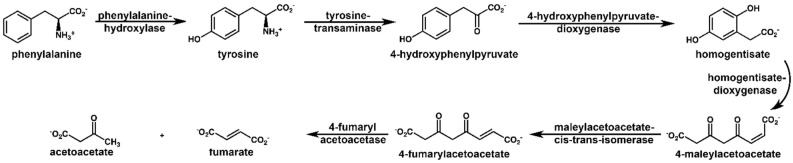
Phenylalanine and tyrosine degradation pathway (KEGG module hsa_M00044); concerning AKU, the critical step is opening the aromatic ring of homogentisate, catalyzed by the enzyme homogentisate dioxygenase (HGD), resulting in the acyclic 4-maleylacetoacetate. In AKU, variants of the HGD gene lead to a dysfunctional enzyme and subsequent substrate accumulation, leading to the formation of a dark pigment (the ochronotic pigment).

Alkaptonuria (AKU; OMIM 203500) is a painful multisystemic disease [1] with a prevalence of one in 250,000, caused by the accumulation of homogentisic acid (HGA). HGA is an intermediate product of phenylalanine and tyrosine degradation. Both amino acids, in physiological conditions, are oxidized to fumaric and acetoacetic acids in a subpathway (KEGG module hsa_M00044) of tyrosine metabolism (see Figure 1). The aromatic ring is opened and oxidized by the hexameric homogentisate-1,2-dioxygenase (HGD) [2]. AKU patients carry homozygous or compound heterozygous variants within the gene coding for HGD. To date, 252 different HGD variants are reported by the HGD mutation database (http://hgddatabase.cvtisr.sk/ (accessed on 7 November 2022)) [3] from the DNA sequencing of approximately 730 AKU patients. HGD variants affect the catalytic activity in multiple ways [4,5,6,7], causing the accumulation of HGA. Such accumulation leads to the formation of a dark pigment (the ochronotic pigment) and the development of alkaptonuria [4,8]; it also triggers inflammatory [9,10,11,12] and secondary amyloidosis [13,14] processes. The kidney removes HGA leading to gram quantities in the urine, which assumes a dark color once in contact with air [15], a process speeded up by adding strong bases. Indeed, alkaptonuria is named after the term Alkapton, coined in 1859 by Boedeker [16] from the Greek alkali “alkali” and kápton, “to gulp down”, after he noticed an unusual reducing property and dark coloring upon the alkalinization of the urine in a patient suffering from AKU. In 1866, ochronosis was described and named for the first time by Virchow [17], who observed a dark pigment under microscopy, which appeared to be ochre in color. The compound responsible for the development of the dark color was identified in 1891 by Baumann and Wolkow [18] as the homogentisic acid. The mechanism leading HGA to ochronotic pigment has been recently elucidated [19] and reported to proceed via radical intermediates to a large, rigid molecule on the nanometer scale. From the third to fourth decade of life, severe consequences of such ochronotic processes are observed, such as valvular heart disease, spondyloarthropathy, or the rupture of ligaments/muscles/tendons.

Sir Archibald Garrod, in 1902, defined AKU as an “inborn error of metabolism” because he noticed the inheritance through which the pathology appeared. Indeed, AKU is one of the first pathologies that confirms the principles of Mendelian recessive inheritance [20]. Nowadays, the (known) countries affected by this disease are only 40, with some areas, for example, Slovakia or the Dominican Republic, showing a much higher frequency than the rest [20]. Based on the results of the SONIA 2 (Suitability of Nitisinone in Alkaptonuria 2) clinical study [21], the European Medicines Agency (EMA) recently approved the use of Orfadin^®^ (nitisinone) for the treatment of adult patients with AKU. Nitisinone, a drug approved by the U.S. Food and Drug Administration for tyrosinemia type 1, can decrease urine HGA by more than 80%, arrest ochronosis, and reduce AKU’s progression rate [22,23,24,25]. Nitisinone is a benzoylcyclohexane-1,3-dione that reversibly inhibits the enzyme before HGD, the 4-hydroxyphenylpyruvate dioxygenase (HPPD), hence reducing the production of homogentisic acid (Figure 1). The effects of nitisinone treatment have been well characterized from a metabolomic point of view [23,26,27,28]. The main side effect of nitisinone administration is the elevation of plasma tyrosine levels analogous to tyrosinemia type 2, which might cause eye pathology in AKU patients [29]; moreover, the effect of nitisinone on already-developed ochronosis is limited [1]. Thus, alternative/adjuvant treatment decreasing HGA without causing tyrosinemia is an urgent need in AKU to be addressed with a better understanding of the mechanisms underlying ochronosis [13] and variability in the disease severity [30]. In this view, untargeted metabolomics of biofluids allows the direct investigation of the involvement and interplay of molecular species in metabolic diseases. Here, we present the untargeted metabolomics study of AKU via nuclear magnetic resonance of urine from a cohort of Italian patients aiming to unravel other molecular mechanisms and molecular species underlying the AKU metabolic disorder. New potential biomarkers beyond homogentisate are suggested, contributing to a more comprehensive molecular signature for AKU and future adjuvant treatment.

## 2. Results and Discussion

### 2.1. NMR Spectroscopy

Urine samples of 34 AKU patients (non-treated) and one under nitisinone administration have been analyzed by NMR experiments, and a total of 61 metabolites have been detected and quantified. However, seven metabolites were excluded because they were drug metabolites or constituents of a vehicle for drug administration (salicylate, mannitol), belonged to microbial metabolism (methanol, methylamine, dimethylamine, trimethylamine), or had unreliable quantification such as urea [31] because suppression of the water NMR signal by pre-saturation may lead to resonant suppression of the urea peak due to proton exchange with the solvent [32]. A control set of 34 samples from healthy individuals with matched age/sex has been similarly characterized. Multivariate analysis, biomarker analysis, and enrichment analysis have been carried out to obtain the whole metabolic profiling of AKU.

**Figure 2:**
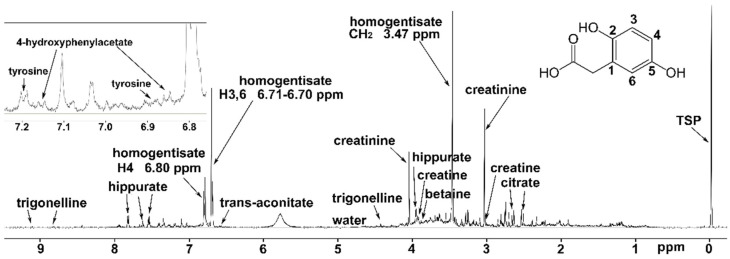
Proton 1D spectrum of a urine sample from the AKU non-treated patient with the lowest HGA concentration (AKU.356, 2.7 mM); even at the lowest concentration, HGA signals dominate the spectrum both upfield (CH_2_ singlet at 3.47 ppm) and downfield (aromatic system from hydrogens 3,6 and 4 at 6.70, 6.71 and 6.80 ppm); at the same time, low concentration metabolites (e.g., tyrosine and 4-hydroxyphenilacetate, see inset) are detected and quantified. Signals of other relevant metabolites among the sixty identified are labeled.

The NMR signals of homogentisate, the hallmark biomarker of AKU, dominate the spectra of AKU samples (Figure 2). HGA is characterized by the sharp singlet of methylene at 3.47 ppm and the aromatic system at 6.70, 6.71, and 6.80 ppm. The latter peaks lie in a region almost free from signals of other metabolites, allowing accurate quantification and high sensitivity for HGA.

AKU samples show the metabolite in the tens of millimolar range (Table 1), orders above all the other molecules (except for creatinine). In contrast, controls show no detectable peaks to the limit of detection (LoD, see Materials and Methods).

### 2.2. Multivariate Analysis

**Figure 3:**
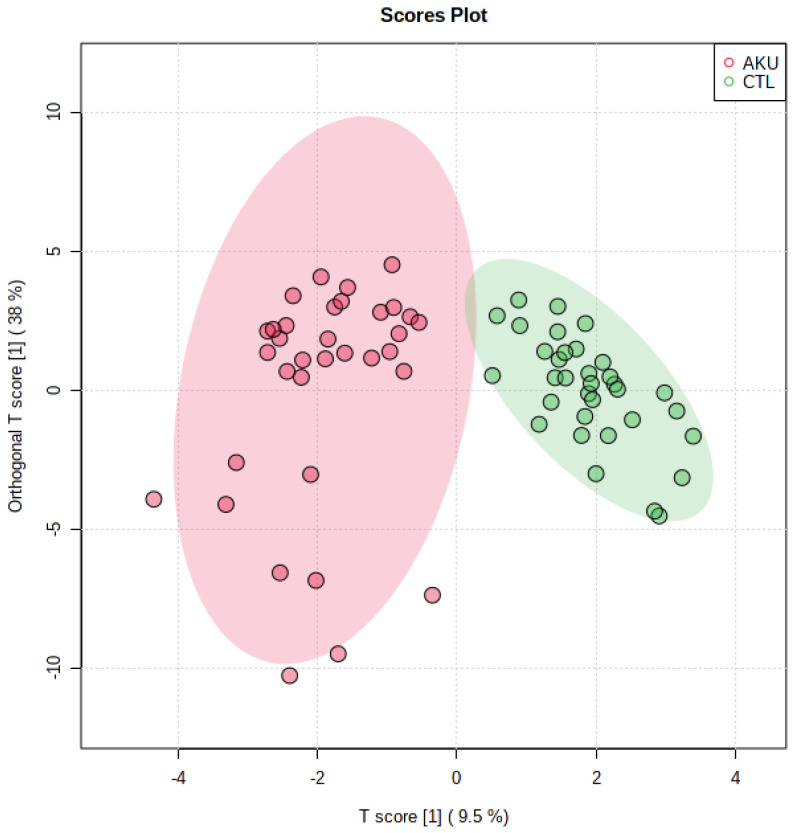
Orthogonal PLS-DA for AKU/CTL groups. An excellent differentiation between groups (T score) is apparent. Intra-group variation (o-*T* score) is very limited in controls; it is also narrow in AKU but for a few points, to be ascribed to the considerable variation in HGA concentrations (see Table 1).

To investigate inter- and intra-group variation, a supervised approach to multivariate dimensionality reduction, such as orthogonal PLS-DA, has been adopted (Figure 3). The *T* score/*o*-*T* score plot shows how AKU and control groups are well differentiated, while intra-group variation is narrow in both groups but for a few points in AKU; here, the variability is to be ascribed to the large excess of HGA in some samples; e.g., of the nine negative *T*-score points, six are the topmost concentrated in HGA with values exceeding 30 mM. Multivariate statistics suggest that the untargeted NMR study has the potential to investigate new potential biomarkers beyond homogentisate, leading to a molecular signature for AKU.

### 2.3. Pathway Analysis

**Figure 4:**
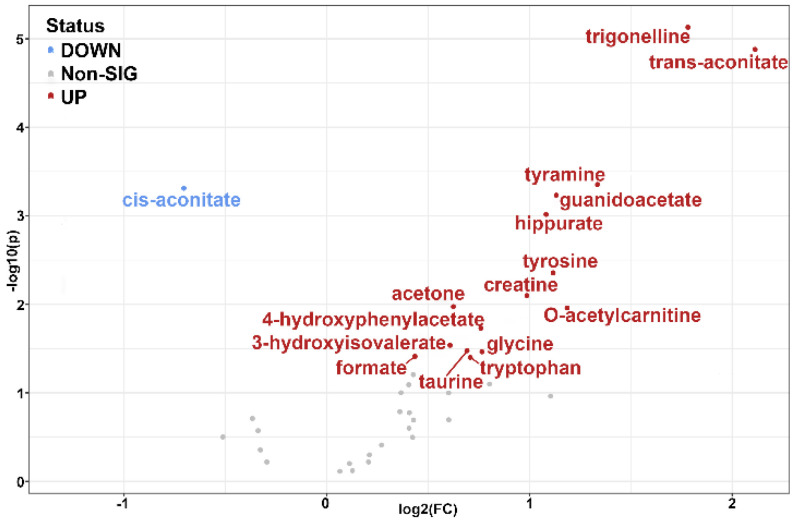
Volcano plot of the fold change vs. *p*-value for the metabolite dataset; the binary logarithm of fold change is used to increase the dynamic range. Significant metabolites (*p* < 0.05) are labeled and colored according to the increase (red) or decrease (blue) for AKU vs. controls. Homogentisate values from Table 2 have been omitted for clarity because of their high range.

The role of metabolites other than HGA in AKU has been evaluated by fold change analysis (Table 2 and Figure 4), and their significance, given their role in metabolic pathways, is discussed in the following paragraphs.

After HGA, the most significant increase in AKU is shown by another acid, the trans-aconitic acid (TAA, log_2_(FC) = 2.11), although to a lower extent, ca. 0.15 mM on average. TAA has been recently reported as one of the top metabolites that significantly increased and associated with laboratory variables indicative of liver and kidney disease severity [33]; in the same study, TAA also resulted among the five metabolites predicting mortality from cirrhosis, making it a significant marker in hepatorenal dysfunction. TAA is also known to play a role in the TCA cycle where it inhibits the aconitase (ACO), the enzyme converting citrate to isocitrate via cis-aconitic acid (CAA) intermediate [34,35,36]. Consistently, cis-aconitate shows a significant decrease in AKU (log_2_(FC) = −0.70).

The impairment is even more evident when looking at the average CAA/TAA ratio, which scores 20 for healthy subjects, and dramatically reduces to four in AKU. The downregulation of the TCA-related metabolites has already been reported for animal models of AKU [28]. When TAA accumulates, the cells clear it through methylation or transport to avoid inhibition and maintain the TCA cycle at work.

Another significant increase in AKU regards trigonelline (log_2_(FC) = 1.78). It is the product of N-methylation of nicotinate (part of B3 vitamin forms) and an endpoint metabolite excreted with the urine. Together with other molecules within one-carbon metabolism, e.g., guanidinoacetate and formate [37] (incremented by log_2_(FC) = 1.13 and 0.44, respectively), it has been suggested to represent the daily turnover and oxidation of amino acids in the lean tissues [38]. Nicotinate has a key role in NAD+ synthesis, where it reacts with phosphoribosyl diphosphate (PPRP) to produce nicotinate ribonucleotide. Nicotinate can be derived from tryptophan, which also significantly increases in AKU (log_2_(FC) = 0.71). Both trigonelline and tryptophane increase maybe then related to nicotinate/nicotinamide metabolism.

*O*-Acetylcarnitine (log_2_(FC) = 1.18) is an acetyl-CoA conversion product that participates in endogenous carnitine homeostasis together with heavier acyl-carnitines and L-carnitine [39]. Such molecules are highly regulated through reabsorption (98%) in the renal tubules and distribution in the tissues via the organic cation/carnitine transporters [40,41]. The increased *o*-acetylcarnitine level is a buffer mechanism between glucose oxidation and fat oxidation, referred to as metabolic flexibility [42,43]. Persistent elevated *o*-acetylcarnitine concentrations may represent a signal of metabolic inflexibility [39]. It has been suggested that acetylcarnitine provides a disposal route for excess acetyl-CoA [44]; accordingly, another acetyl-CoA product, acetone, is increased in AKU. Interestingly, the aforementioned study on the AKU animal model [28] demonstrated an increase in the range of acetylated conjugates, indicating increased acetyl-CoA activity. Such collective findings allow speculation that acetyl-CoA misregulation may be linked to TCA cycle malfunction.

Hippurate (HIP, log_2_(FC) = 1.08) is synthesized in the liver through the conjugation of glycine and benzoate; the latter is produced by gut bacteria [45] from the degradation of unabsorbed phenylalanine to 3-phenylpropionic acid (PPA), which is normally esterified with CoA in the liver and oxidized to benzoyl-CoA in a reaction catalyzed by mitochondrial Medium-chain acyl-Coenzyme A dehydrogenase (MCAD). Benzoyl-CoA is rapidly converted to hippuric acid by condensation with glycine and is excreted in the urine [46]. Although hippurate participates in correcting metabolic acidosis [47], a condition triggered by metabolic complications in patients with Alkaptonuria [48], it must be considered that the diet is the primary source of the benzoate precursor [49]; further investigation is then needed on this metabolite.

The glycine metabolism pathway shows a significant increase in the glycine/guanidinoacetate/creatine axis. Creatine is synthesized endogenously from arginine and glycine through arginine-glycine amidinotransferase (AGAT) and guanidinoacetate methyltransferase (GAMT) [50,51]. Together with one-carbon species, it is referred to as SGOC metabolic unit (Serine, Glycine, and One-Carbon). An increase in urinary creatine excretion, as well as for taurine, has been observed in destructive and inflammatory muscle disorders [52,53]. The increase in creatine, its precursor guanidinoacetate, and taurine may be ascribed to muscle damage from AKU condition and be connected to the higher risk of muscle complications that AKU patients carry with respect to others with osteoarthritis disease [54,55].

**Figure 5:**
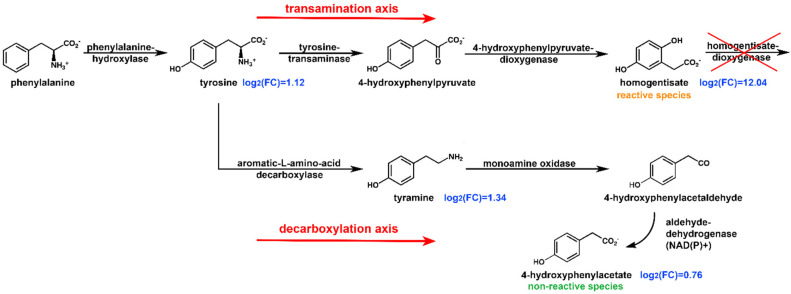
Alternative ways for tyrosine degradation via transamination (top) and decarboxylation (bottom) and their behavior in AKU (metabolites fold change is reported in blue color as binary logarithm). The transamination axis is interrupted at the homogentisate dioxygenase step, leading to a dramatic increase in homogentisate concentrations in urine samples. Tyrosine levels are also doubled as a consequence. The impairment of the transamination axis leads to a stimulation of the decarboxylation axis with a similar increase of tyramine and 4-hydroxyphenylacetate levels in urine.

The phenylalanine and tyrosine degradation pathway is represented by four metabolites: tyrosine, homogentisate, tyramine, and 4-hydroxyphenylacetate (HPAA). Deficiency in homogentisate dioxygenase in AKU causes a tremendous fold change in HGA urine levels (log_2_(FC) = 12.04) as described in the introduction, also reflected in tyrosine level increment (log_2_(FC) = 1.12) due to clearance impairment of the transamination axis (Figure 5). Transamination is not the only way of tyrosine degradation: as it has been shown that in kidneys, decarboxylation may occur by aromatic-1-amino decarboxylase (AADC) producing tyramine [56]. Because of its high sensitivity to oxidation by MAO, endogenous tyramine levels are very low; indeed, it is one of a group of biogenic amines (BAs) which are referred to as “trace amines” [57,58], together with β-phenylethylamine, tryptamine, p-octopamine, and some of their metabolites. Our analysis showed a log_2_(FC) = 1.34 for tyramine (a value even superior to that of tyrosine), inferring upregulation of the decarboxylation axis. A tyramine increment has also been observed for Tyrosinemia type II [59], where, similarly, the pathway is interrupted at the step catalyzed by tyrosine transaminase. Interestingly, AADC has long been regarded as an unregulated enzyme. Still, extensive studies showed it is regulated both pre- and post-translation [60], e.g., AADC has several conserved protein kinase A (PKA) and protein kinase G (PKG) recognition sites [61,62]. Consistently with tyramine increment, its oxidative deaminated product 4-hydroxyphenylacetate also shows a significant increase (log_2_(FC) = 0.76). Its production is catalyzed by aldehyde dehydrogenase (ALDH), with ALDH3 isozyme preferentially oxidizing aromatic aldehyde substrates. Eventually, a saturation of the transamination axis pathway leads to urine level increments of other aromatic species.

Eventually, metabolomic profiling identified multiple metabolic routes (Table 2) as influenced by AKU; however, the sole NMR analysis does not cover the full metabolome, and different approaches are needed to expand the picture. In this view, the only other study on untreated AKU to date was conducted by mass spectrometry analysis on a mouse model [28], and the results are worth comparing. Both studies agree on the elevated levels of tyrosine metabolites, particularly 4-hydroxyphenylacetate, although only NMR reveals a significative increment of tyrosine itself and tyramine. Mass spectroscopy additionally detects HGA elimination metabolites (e.g., HGA-sulfate and -glucuronide), while NMR reports SGOC metabolites increase. The two studies agree on the misregulation of the TCA cycle and increased acetyl-CoA availability. Indeed, data integration from different models/techniques contributes to expanding the picture in a rare disease such as alkaptonuria and may overcome some limitations. For example, renal metabolism may contribute to the urine metabolomic profile [63], suggesting the inclusion of further metabolomics analysis of, e.g., intact serum/plasma [64]. Moreover, damage to joints in alkaptonuria appears to be correlated with HGA or its metabolites directly attacking structural proteins by, e.g., free radical attack [19], suggesting further molecular studies directed at the damaged tissues are needed.

### 2.4. NMR Metabolic Profile of a Nitisinone-Treated Patient

**Figure 6:**
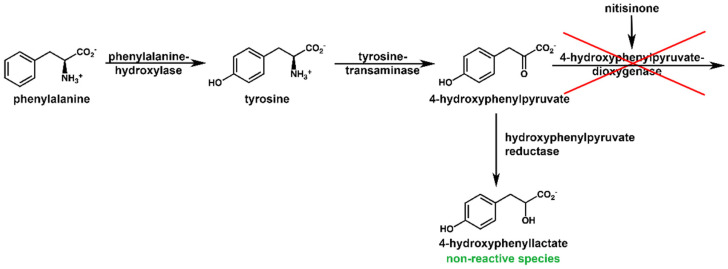
Inhibition of 4-hydroxyphenylpyruvate-dioxygenase by nitisinone makes two new metabolites detectable in AKU urine, the 4-hydroxyphenylpyruvate and its reduction product 4-hydroxyphenyllactate. The latter has been proved by NMR to be a non-reactive species when exposed to alkali.

One patient, excluded from the analysis above, was in treatment with nitisinone, a drug inhibiting 4-hydroxyphenylpyruvate-dioxygenase (HPPD), thus limiting the step that brings to homogentisate and subsequently its accumulation (Figure 6). Despite the single-point measure, significant observations can be reported. HGA level for non-treated patients ranges from 3 mM to 65 mM, while for the treated one, the value is as low as 0.13 mM, as expected for HPPD blockade. The other metabolites involved, tyrosine, tyramine, and 4-hydroxyphenylacetate, show levels similar to non-treated patients. This is consistent with nitisinone action, stopping the pathway just one enzymatic step before HGD and not changing the upstream dysregulation in AKU. The other significant change in the treated patient is the sudden appearance of 4-hydroxyphenyllactate (HPLA, from non-detectable to a remarkable 3.5 mM) and of its precursor 4-hydroxyphenylpyruvate (HPPA, from non-detectable to 0.4 mM). Such behavior of HPLA and HPPA, observed in our NMR analysis of urine of a nitisinone-treated patient, follows that observed for serum in the nitisinone administration regime [65] by LC/MS analysis.

### 2.5. Reactivity of HPAA and HPLA

Both HGD and HPPD enzyme activity impairment has demonstrated significant increment of alternative tyrosine pathway routes toward 4-hydroxyphenylacetate and 4-hydroxyphenyllactate. Their increment in urine, although to a much lesser extent with respect to homogentisate, has not been evaluated for reactivity similar to their parent compound homogentisate in aciduria studies. Both HPAA and HPLA have been tested under alkaline conditions following the procedure from Bernini et al. [19]. NMR analysis showed neither change in molecular structure nor in concentration (See Supplementary Material, Figures S1–S3), inferring the two compounds to be insensitive to full deprotonation or decarboxylation. Such a condition, on the contrary, leads homogentisate to react and to discoloration [19]. Such evidence confirms that the two metabolites, as expected, do not contribute to ochronosis-like processes.

## 3. Materials and Methods

### 3.1. Biological Samples

Adult patients (*n* = 35, 19 females and 16 males, aged 53 on average) were studied after the clinical diagnosis of AKU was established. None of the patients was under a specific diet or treatment, but one under the administration of nitisinone, not included in the statistics. Age-matched controls of both sexes with no arthropathies or metabolic disorders were also analyzed (*n* = 34). Urine samples were obtained from patients and control subjects after overnight fasting and promptly stored at −80 °C. Patients and controls gave written informed consent before inclusion. The whole study was conducted following the approval of the Siena University Hospital Ethics Committee. The informed consent and protocols conformed to the standards set by the latest revision of the Declaration of Helsinki.

### 3.2. Sample Preparation

Dipotassium hydrogen phosphate (K_2_HPO_4_), monopotassium phosphate (KH_2_PO_4_), and trimethylsilylpropionic acid-d4 (TSP) have been purchased from Merck (MerckKGaA, Darmstadt, Germany). Deionized water was purified using a Milli-Q^®^ System from Millipore (Burlington, MA, USA). A stock solution of phosphate buffer 333 mM (PB) was prepared from K_2_HPO_4_ and KH_2_PO_4_, and pH was adjusted to 7.0 with NaOH 1M. NMR samples were prepared with 300 µL of urine, 30 µL TSP and 270 µL PB to final concentrations of 0.5 mM TSP and 150 mM PB. NaN_3_ 0.2% (*p*/*p*) was added to samples to prevent microbial proliferation.

### 3.3. ^1^H NMR Spectroscopy

All experiments were performed on a Bruker Avance™ 600 spectrometer (Bruker Biospin AG, Fällanden, Switzerland) operating at 14.1 T. An optimized-PURGE [66] pulse sequence was used. The NMR data were processed with the TopSpin 4.0.8 software and analyzed with Chenomx 9.02 (Edmonton, AB, Canada). All the 1D proton spectra were acquired with a spectral width of 16 MHz and 32 scans digitalized over 32k points and RG of 128. Solvent signal removal was achieved with pre-saturation power of 55 dB during repetition delay (4s). The following metabolites were identified and quantified (see Supplementary Table S1 for details): 1-methylnicotinamide, 2-aminoadipate, 2-furoylglycine, 2-hydroxyisobutyrate, 3-aminoisobutyrate, 3-hydroxyisovalerate, 3-indoxylsulfate, 4-hydroxybutyrate, 4-hydroxyphenylacetate, 4-hydroxyphenyllactate, 4-hydroxyphenylpyruvate, acetate, acetoacetate, acetone, cis-aconitate, trans-aconitate, alanine, betaine, carnitine, choline, citrate, creatine, creatinine, dimethylamine, erythritol, ethanol, ethanolamine, formate, galactarate, glucose, glutamine, glycine, glycolate, guanidinoacetate, hippurate, homogentisate, hypoxanthine, myo-inositol, isobutyrate, isoleucine, lactate, mannitol, methanol, methylamine, N-phenylacetylglycine, *O*-acetylcarnitine, pyroglutamate, salicylate, succinate, sucrose, taurine, trigonelline, trimethylamine, trimethylamine N-oxide, tryptophan, tyramine, tyrosine, uracil, urea, valine, xylose.

The limit of detection (LoD) for HGA in urine in the present experimental conditions has been evaluated by adding known amounts of the acid from a stock solution to control samples. An added concentration as low as 5 µM (0.8 µg/mL) raises detectable peaks at 6.70/6.71 ppm (see Supplementary Materials). None of the controls showed such peaks indicating HGA is absent or negligible in control vs. AKU. Statistical analysis and pathway analysis have been performed using MetaboAnalyst 5.0 [67].

## 4. Conclusions

Metabolic research on alkaptonuria and nitisinone treatment usually focuses on the metabolic route involving homogentisate because its accumulation triggers ochronosis and subsequent disease development. In search of a more comprehensive molecular signature of AKU, we have carried out the first metabolome-wide comparison of untreated AKU patients versus non-AKU subjects. The NMR untargeted study suggests other metabolic pathways to be misregulated in connection with the disease. Noticeably, the SGOC metabolic unit, which includes serine, glycine, and one-carbon metabolism, is particularly affected. SGOC metabolism integrates nutritional status from amino acids, glucose, and vitamins, and generates diverse outputs, such as biosynthesis, the maintenance of redox status, and the substrates for methylation reactions. The impairment of hepatorenal functions is suggested by a large increase of biomarkers such as trans-aconitate and hippurate, while elevated acetylcarnitine and acetone concentrations may represent a signal of excess acetyl-CoA possibly linked to TCA cycle malfunction. A major involvement of phenylalanine and tyrosine obviously emerges in AKU for a reason described at the beginning of the section. Nonetheless, new observations arise from our study. In addition to the concentration increase expected for the species belonging to the transamination axis (Figure 5), a significant increase is reported for the species belonging to the decarboxylation axis in non-treated AKU patients. Like an upstream overflow mechanism, the decarboxylation axis activates to help drain the transamination axis from homogentisate in exchange for 4-hydroxyphenylacetate, an endpoint, non-reactive species. Such a mechanism will be worth further investigation from the genomics and proteomics point of view, as enzymatic activity modulation can possibly be exploited for alternative/adjuvant treatments aiming to decrease HGA accumulation.

## Figures and Tables

**Table 1:** The concentration of homogentisate in the urine of AKU patients.

Patient Code	C_HGA_ (mM)	S.D.	Patient Code	C_HGA_ (mM)	S.D.
AKU.303	11.33	0.04	AKU.413	21.36	0.17
AKU.304	14.07	0.08	AKU.414	52.98	0.35
AKU.305	11.22	0.04	AKU.418	15.80	0.03
AKU.338	14.89	0.03	AKU.420	14.49	0.13
AKU.350	37.25	0.12	AKU.423	11.48	0.08
AKU.356	2.77	0.02	AKU.425	33.69	0.29
AKU.364	17.12	0.14	AKU.426	38.77	0.38
AKU.367	16.17	0.13	AKU.427	24.42	0.26
AKU.369	24.22	0.26	AKU.430	10.78	0.02
AKU.370	11.19	0.08	AKU.433	13.38	0.02
AKU.371	35.77	0.37	AKU.434	14.04	0.02
AKU.379	21.36	0.15	AKU.435	17.11	0.02
AKU.381	16.12	0.12	AKU.436	15.51	0.02
AKU.385	12.38	0.11	AKU.443	64.62	0.08
AKU.390	23.01	0.17	AKU.447	34.41	0.06
AKU.410	12.19	0.08	AKU.449	15.88	0.03
AKU.411	23.39	0.03	AKU.450	15.28	0.02

**Table 2:** Fold changes for metabolites in AKU showing a p-value lower than 0.05 are reported (see Figure 4 for a graphical representation). Fold change is reported as a binary logarithm, and the direction of change is reported as arrows; metabolites are assigned to the relevant pathway.

Metabolite	Log_2_(FC)		Up/Down	Pathway
Homogentisate ^a^	12.04	*p* < 10^−13^	↑	Phenylalanine and tyrosine metabolism
trans-Aconitate	2.11	*p* < 0.00001	↑	TCA cycle
Trigonelline	1.78	*p* < 0.00001	↑	SGOC ^b^ metabolic unit
Tyramine	1.34	*p* < 0.0001	↑	Phenylalanine and tyrosine metabolism
*O*-Acetylcarnitine	1.18	*p* < 0.01	↑	Acetyl-CoA conversion
Guanidinoacetate	1.13	*p* < 0.0001	↑	SGOC metabolic unit
Tyrosine	1.12	*p* < 0.001	↑	Phenylalanine and tyrosine metabolism
Hippurate	1.08	*p* < 0.0001	↑	Modulator of metabolic acidosis (MAC)
Creatine	0.99	*p* < 0.001	↑	SGOC metabolic unit
2-Furoylglycine	0.80	*p* < 0.01	↑	Minor fatty acid metabolite
4-Hydroxyphenylacetate	0.76	*p* < 0.01	↑	Phenylalanine and tyrosine metabolism
Glycine	0.76	*p* < 0.05	↑	SGOC metabolic unit
Tryptophan	0.71	*p* < 0.05	↑	Tryptophan metabolism
Taurine	0.69	*p* < 0.05	↑	Taurine metabolism
Acetone	0.62	*p* < 0.01	↑	Acetyl-CoA conversion
3-Hydroxyisovalerate	0.61	*p* < 0.05	↑	Leucine degradation
Formate	0.44	*p* < 0.05	↑	SGOC metabolic unit
cis-Aconitate	−0.70	*p* < 0.0001	↓	TCA cycle

^a^ FC of homogentisate has been evaluated using the experimentally determined LoD value of 5 µM for control samples since no peaks arise from the baseline background noise for such samples. ^b^ SGOC: serine, glycine, and one-carbon metabolism.

## Supplementary Materials

The following supporting information can be downloaded at: https://www.mdpi.com/article/10.3390/ijms232415805/s1.
