# Supplementary material for: Untargeted NMR Metabolomics Reveals Alternative Biomarkers and Pathways in Alkaptonuria
Source: Int J Mol Sci. 2022 Dec 13;23(24):15805. doi: 10.3390/ijms232415805 (PMC9779518; doi:10.3390/ijms232415805)
Supplement: Supplementary file 1 [file ijms-23-15805-s001.zip › ijms-2055328-supplementary.pdf]

Untargeted NMR Metabolomics Reveals Alternative Biomarkers and Pathways in Alkaptonuria
Supplementary material

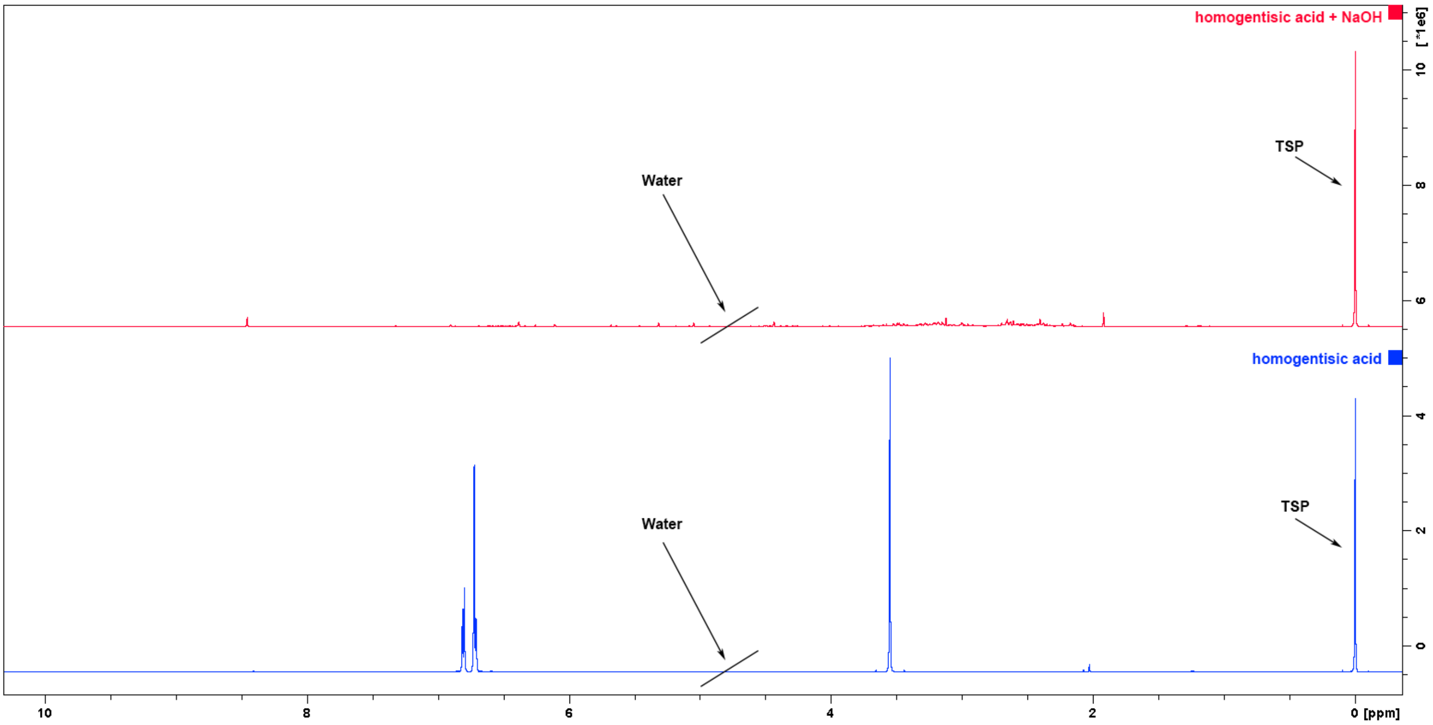

Figure S1. Proton 1D spectra of homogentisic acid (blue) (CH_2 singlet at 3.47 ppm and the aromatic system from hydrogens 3,6 and 4 at 6.70, 6.71 and 6.80 ppm) and of homogentisic acid added with 5 eq. of NaOH (red). In an alkaline environment, HGA signals disappear rapidly, and only residual signals arise from the reacted solution (see [19]).

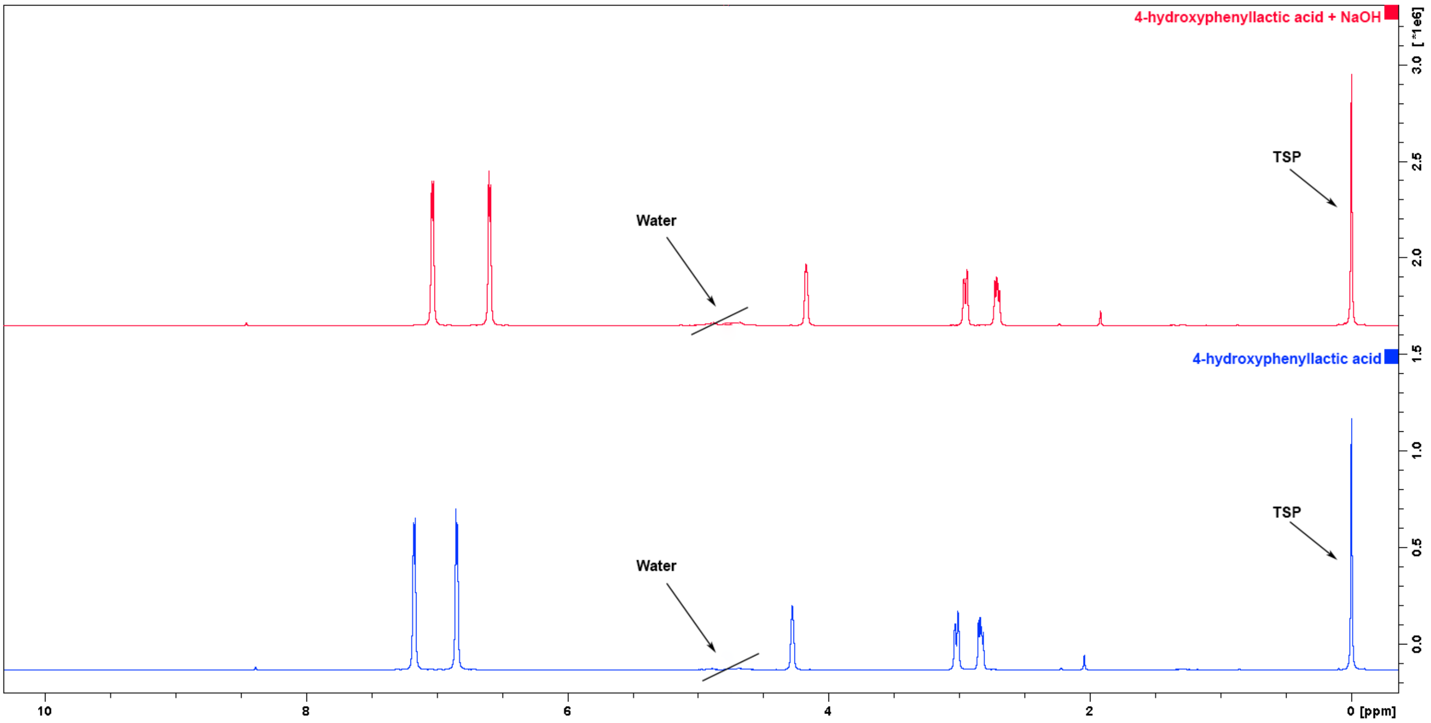

Figure S2. Proton 1D Spectra of 4-hydroxyphenyllactic acid (blue) (two clusters from the hydrogens of CH_2 at 2.84 ppm and 3.02 ppm, the CH quadruplet at 4.28 ppm and the aromatic system from hydrogens 3,5 and 2,6 at 6.85 and 7.17 ppm) and of 4-hydroxyphenyllactic acid added with 5 eq. of NaOH (red). Unlike HGA, no change occurs upon alkalinization (except for the shift due to the pH increase), inferring no reaction takes place.

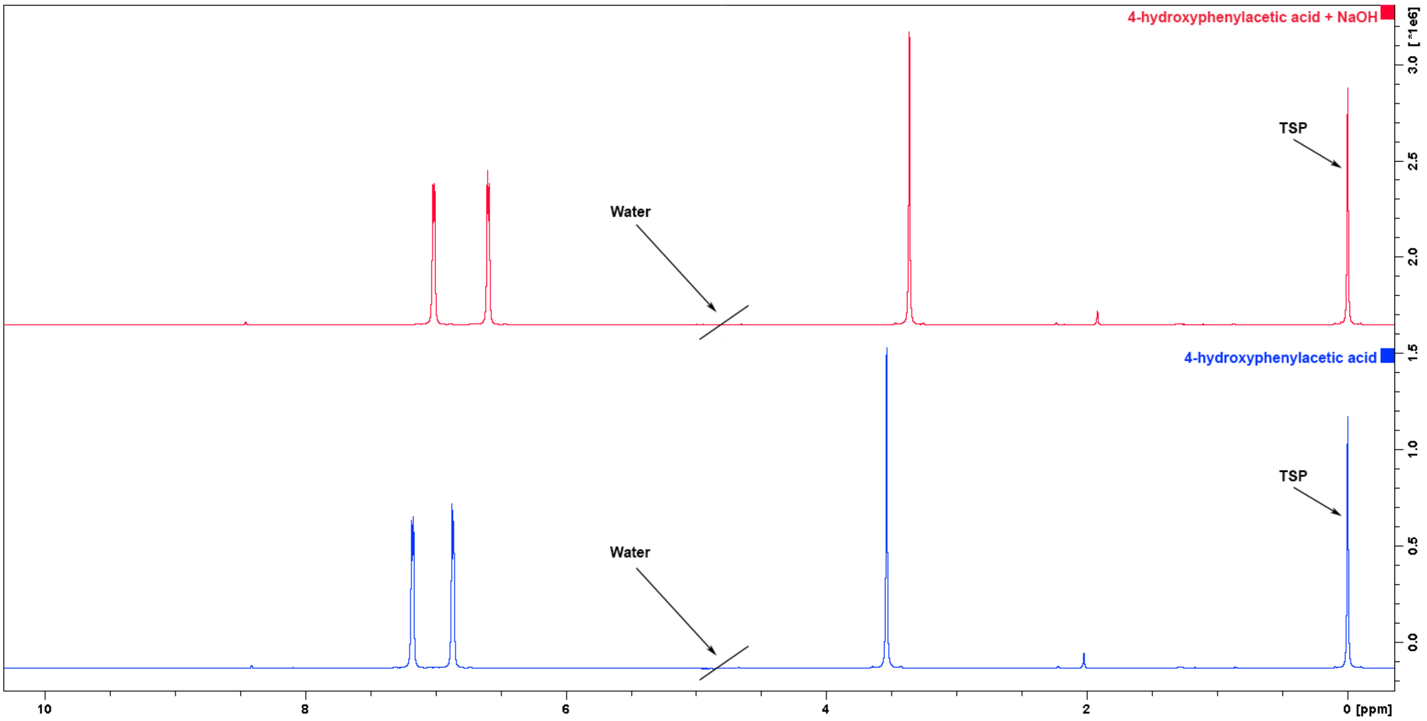

Figure S3. Proton 1D Spectra of 4-hydroxyphenylacetic acid (blue) (CH_2 singlet at 3.54 ppm and the aromatic system from hydrogens 3,5 and 2,6 at 6.87 and 7.18 ppm) and of 4-hydroxyphenylacetic acid added with 5 eq. of NaOH (red). Unlike HGA, no change occurs upon alkalinization (except for the shift due to the pH increase), inferring no reaction takes place.

Table S1. Metabolites identified by NMR analysis of urine samples with direction and significance of fold change for AKU concentrations vs. controls. HMDB codes are also reported. Seven compounds were excluded because they are drug metabolites, constituents of a drug administration vehicle, belong to microbial metabolism, or have unreliable quantification, such as urea.

Metabolite	FC (AKU/CNTR)	HMDB Code
1-Methylnicotinamide	Not significative	HMDB00699
2-Aminoadipate	Not significative	HMDB00510
2-Furoylglycine	Increased	HMDB00439
2-Hydroxyisobutyrate	Not significative	HMDB00729
3-Aminoisobutyrate	Not significative	HMDB03911
3-Hydroxyisovalerate	Increased	HMDB00754
3-Indoxylsulfate	Not significative	HMDB00682
4-Hydroxybutyrate	Excluded	HMDB00710
4-Hydroxyphenylacetate	Increased	HMDB00020
4-Hydroxyphenyllactate	Increased only under nitisinone	HMDB00755
Acetate	Not significative	HMDB00042
Acetoacetate	Not significative	HMDB00060
Acetone	Increased	HMDB01659
cis-Aconitate	Decreased	HMDB00072
trans-Aconitate	Increased	HMDB00958
Alanine	Not significative	HMDB00161
Betaine	Not significative	HMDB00043
Carnitine	Not significative	HMDB00062
Choline	Not significative	HMDB00097
Citrate	Not significative	HMDB00094
Creatine	Increased	HMDB00064
Creatinine	Not significative	HMDB00562
Dimethylamine	Not significative	HMDB00087
Erythritol	Not significative	HMDB02994
Ethanol	Excluded	HMDB00149
Ethanolamine	Not significative	HMDB00149
Formate	Increased	HMDB00142
Galactarate	Not significative	HMDB00639
Glucose	Excluded	HMDB00122
Glutamine	Not significative	HMDB00641
Glycine	Increased	HMDB00123
Glycolate	Not significative	HMDB00115
Guanidinoacetate	Increased	HMDB00128
Hippurate	Increased	HMDB00714
Homogentisate	Increased	HMDB00130
Hypoxanthine	Not significative	HMDB00157
Myo-inositol	Not significative	HMDB00211
Isobutyrate	Not significative	HMDB01873
Isoleucine	Not significative	HMDB00172
Lactate	Not significative	HMDB00190
Mannitol	Not significative	HMDB00765
Methanol	Excluded	HMDB01875
Methylamine	Not significative	HMDB00164
N-phenylacetylglycine	Not significative	HMDB00821
O-acetylcarnitine	Increased	HMDB00201
Pyroglutamate	Not significative	HMDB00267
Salicylate	Excluded	HMDB01895
Succinate	Not significative	HMDB00254
Sucrose	Not significative	HMDB00258
Taurine	Increased	HMDB00251
Trigonelline	Increased	HMDB00875

Trimethylamine	Not significative	HMDB00906
Trimethylamine N-oxide	Not significative	HMDB00925
Tryptophan	Increased	HMDB00929
Tyramine	Increased	HMDB00306
Tyrosine	Increased	HMDB00158
Uracil	Excluded	HMDB00300
Urea	Excluded	HMDB00294
Valine	Not significative	HMDB00883
Xylose	Not significative	HMDB00098
4-hydroxyphenylpyruvate	Increased only under nitisinone	HMDB00707
